# High Solubilization and Controlled Release of Paclitaxel Using Thermosponge Nanoparticles for Effective Cancer Therapy

**DOI:** 10.3390/pharmaceutics13081150

**Published:** 2021-07-27

**Authors:** Jin Sil Lee, Hyeryeon Oh, Daekyung Sung, Jin Hyung Lee, Won Il Choi

**Affiliations:** 1 Center for Convergence Bioceramic Materials, Convergence R&D Division, Korea Institute of Ceramic Engineering and Technology, 202, Osongsaengmyeong 1-ro, Osong-eup, Heungdeok-gu, Cheongju 28160, Korea; jslee92@kicet.re.kr (J.S.L.); hyeryeon.oh@kicet.re.kr (H.O.); dksung@kicet.re.kr (D.S.); leejinh1@kicet.re.kr (J.H.L.); 2 School of Materials Science and Engineering, Gwangju Institute of Science and Technology, Cheomdan-gwagiro 123, Buk-gu, Gwangju 61005, Korea

**Keywords:** thermosponge nanoparticle, poorly water-soluble drug, paclitaxel, high drug loading amount, controlled release, cancer

## Abstract

Cancer, which is a leading cause of death, contributes significantly to reducing life expectancy worldwide. Even though paclitaxel (PTX) is known as one of the main anticancer drugs, it has several limitations, including low solubility in aqueous solutions, a limited dosage range, an insufficient release amount, and patient resistance. To overcome these limitations, we suggest the development of PTX-loaded thermosponge nanoparticles (PTX@TNP), which result in improved anticancer effects, via a simple nanoprecipitation method, which allows the preparation of PTX@TNPs with hydrophobic interactions without any chemical conjugation. Further, to improve the drug content and yield of the prepared complex, the co-organic solvent ratio was optimized. Thus, it was observed that the drug release rate increased as the drug capacity of PTX@TNPs increased. Furthermore, increasing PTX loading led to considerable anticancer activity against multidrug resistance (MDR)-related colorectal cancer cells (HCT 15), implying a synergistic anticancer effect. These results suggest that the solubilization of high drug amounts and the controlled release of poorly water-soluble PTX using TNPs could significantly improve its anticancer therapy, particularly in the treatment of MDR-p-glycoprotein-overexpressing cancers.

## 1. Introduction

Cancer is a leading cause of death and a significant contributor to reducing life expectancy worldwide [1]. Current cancer treatments include a variety of strategies, namely, radiation therapy, surgery, chemotherapy, immunotherapy, and targeted therapy [2]. The commencement of the use of the major cancer treatment, chemotherapy, which involved the use of nitrogen mustards and antifolate drugs for the first time, occurred in the 1940s [3]. Currently, several anticancer drugs, including alkylating agents, hormones, antibiotics, antimetabolites, and natural products, have been developed for the treatment of various cancers. Specifically, paclitaxel (PTX), which belongs to one of the main sub-classes of anticancer drugs, the taxanes [4], has shown the ability to successfully suppress cancer growth against a wide range of solid tumors in clinical trials. Notwithstanding its significant anticancer ability, PTX is characterized by a low solubility in aqueous solutions, short biological half-life, and incomplete release kinetics from its formulations. It also leads to hypersensitivity reactions [4,5].

Multidrug resistance (MDR) is an additional problem in cancer therapy with PTX [4,5]. This effect is frequently mentioned as one of the key reasons for the failure of chemotherapy treatment. Resistance to chemotherapeutics may be traced to several mechanisms, such as extended drug efflux, which results in decreased drug uptake, DNA repair process activation along with the detoxifying system, apoptosis suppression, and altered expression of drugs [6,7]. Between two transporter superfamilies, solute carrier (SLC) transporters and ATP-binding cassette (ABC) transporters, the activity of SLC is generally influx, and that of ABC is efflux [8]. A common mechanism of MDR is related to the expression of efflux transporters, such as p-glycoprotein (p-gp). In normal cells, p-gp pumps harmful chemical agents out of the cell, whereas in cancer cells, overexpressed p-gp greatly increases the outflow of anticancer drugs. Thus, the result is a decrease in the intracellular accumulation of therapeutic drugs and unsuccessful cancer treatment [9,10,11,12,13]. Owing to these limitations, PTX is restricted in its clinical applications.

Therefore, to overcome the MDR effect associated with PTX and enhance its therapeutic index, there is a need for other PTX formulations that show improved solubility in aqueous environments. In this regard, numerous carriers, including microspheres, nanoparticles, liposomes, noisomes, PTX-conjugated polymers, and micelles, have been studied to the end of improving the solubility of PTX [5,14,15,16]. Additionally, to combat MDR effects, the co-administration of p-gp inhibitors with anticancer drugs has led to improved intracellular drug uptake [11,17,18,19]. Further, to improve the PTX dosage, the characteristics of carriers have been modified via self-assembly processing and the modification of properties such as the molecular weight of the polymer and the drug loading content by influencing the degradation of the polymer, diffusive processes, and drug dispersion. It has also been demonstrated that the rate of drug release can be boosted by increasing the amount of the drug loaded in the carrier and by using a polymer with a lower molecular weight in the carrier [20,21,22]. However, the optimization of the loading amounts of PTX in carriers for efficient high-lipophilic PTX release kinetics under biological conditions has not yet been reported. 

**Figure 1:**
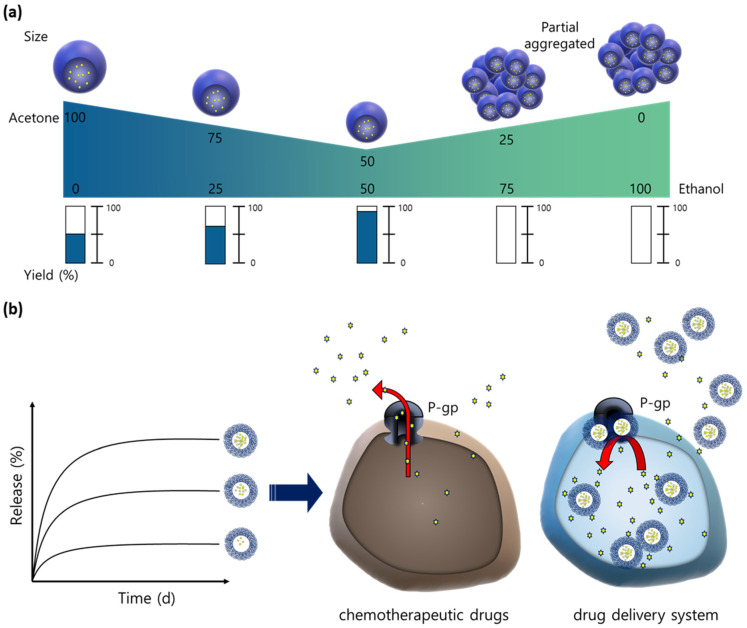
(**a**) Scheme of the characteristics and production yield of paclitaxel (PTX)–loaded TNPs (PTX@TNP) prepared using co–solvents at various ratios; (**b**) release profiles of PTX from TNPs with different loading contents, and mechanism of PTX@TNP–based chemotherapy in MDR-related colorectal cancer cells.

Therefore, in this study, we developed an optimized process for the preparation of PTX-loaded thermosponge nanoparticles (PTX@TNPs) to the end of improving the solubility and release kinetics of the poorly water-soluble PTX, via a simple nanoprecipitation method with various ratios of co-organic solvents. This resulted in effective chemotherapeutic effects in MDR-related colorectal cancer cells (Figure 1). Further, the physicochemical properties of the nanoparticles were analyzed with varied PTX loading amounts into the TNPs. Drug release behaviors were measured in a biological buffer, and the in vitro anticancer effect of the as-produced material was analyzed using a p-gp-overexpressing colorectal cancer cell line (HCT 15).

## 2. Materials and Methods

### 2.1. Materials

PTX was purchased from LC Laboratories (Woburn, MA, USA). Pluronic F127, acetone, and ethanol were obtained from Sigma-Aldrich (St. Louis, MO, USA). Poly (D,L-lactide) with a carboxyl end group (PLA-COOH, MW: 18 kDa) was purchased from Durect (Cupertino, CA, USA). Tween 80 was purchased from TCI (Chuo-ku, Tokyo, Japan), HycloneTM deionized water (DIW) and phosphate-buffered saline (PBS) were obtained from Cytiva (Marlborough, MA, USA), and acetonitrile (HPLC grade) was obtained from Honeywell (Charlotte, NC, USA). The colorectal cancer cell line (HCT 15) was purchased from the American Type Culture Collection (ATCC, Rockville, MD, USA). Fetal bovine serum (FBS), Roswell Park Memorial Institute (RPMI) 1640 medium, trypsin, and antibiotic-antimycotic (AA) were obtained from Thermo Fisher Scientific (Waltham, MA, USA). Cell Counting Kit-8 (CCK-8) was purchased from Dojindo Laboratories (Kumamoto, Japan). 

### 2.2. Preparation of PTX@TNPs with Various Amounts of PTX

PTX@TNPs were prepared without any chemical conjugation, by modifying a previously reported method [23]. To encapsulate various amounts of PTX into TNPs, PTX (1, 5, 10, and 20 mg) was solubilized in acetone and mixed with PLA (10 mg) for 1 h, followed by mixing with Pluronic F127 (200 mg) under rotary shaking for 2 h at 25 °C. Thereafter, to increase the loading amounts and production yield of PTX inside the TNPs, co-solvents (acetone/ethanol) at various ratios (100:0, 75:25, 50:50, 25:75, and 0:100) were used to ensure a higher solubilization of PTX. PTX (5, 10, 15, and 18 mg) was solubilized in the co-solvents, mixed with PLA (10 mg) for 1 h, and subsequently mixed with Pluronic F127 (200 mg) under rotary shaking for 2 h at 25 °C. After that, the mixture was added dropwise to DIW (5 mL) at 530 rpm, and the solvents were removed in a fume hood overnight. Finally, PTX@TNPs were centrifuged at 2500 rpm for 10 min using an Amicon Ultra-15 centrifugal filter (300 kDa molecular weight cut-off (MWCO); Merck Millipore, Burlington, MA, USA) to remove the unloaded drugs. To ensure a higher degree of purification, further filtration was performed using a sterile 0.2 µm syringe filter. 

The physicochemical properties of PTX@TNPs, such as the hydrodynamic diameter, polydispersity index (PDI), and surface charge, were measured using an electrophoretic light scattering spectrophotometer (ELS-Z2, Otsuka Electronics Co., Tokyo, Japan) at 37 °C. Additionally, loading content (L.C) and efficiency (L.E) were analyzed through HPLC and calculated with previously reported equations [24]

$$\begin{array}{c} Loaidng\;content\;\left(L.C\right)\\ =\frac{\left(weight\;of\;the\;feeding\;drug-weight\;of\;unloaded\;drug\right)}{weight\;of\;core\;of\;TNP\;}\;\times \;100 \end{array}$$

$$\begin{array}{c} Loading\;efficiency\;\left(L.E\right)\\ =\frac{weight\;of\;the\;feeding\;drug-weight\;of\;unloaded\;drug}{weight\;of\;the\;feeding\;drug}\;\times \;100 \end{array}$$

and the yield of PTX@TNP was calculated by measuring its weight via freeze drying, as follows:

$$\mathrm{Yield}\;\left(\%\right)=\frac{\mathrm{Initial}\;\mathrm{weight}\;\mathrm{of}\;\mathrm{PTX}@\mathrm{TNP}\;-\;\mathrm{Weight}\;\mathrm{of}\;\mathrm{PTX}@\mathrm{TNP}\;\mathrm{after}\;\mathrm{freeze}\;\mathrm{drying}}{\mathrm{Initial}\;\mathrm{weight}\;\mathrm{of}\;\mathrm{PTX}@\mathrm{TNP}}\;\times \;100$$

### 2.3. In Vitro Drug Release Profiles

The drug release behavior of TNP was determined as previously reported [25,26,27,28]. A PTX@TNP solution (1 mL, 200 μg of PTX) was added to a Float-A-Lyzer G2 dialysis device (100 kDa MWCO, Spectra/Por Dialysis Membrane, Repligen, Waltham, MA, USA) and then immersed in 10 mL of biological solution (PBS, pH = 7.4) containing 0.5% *v*/*v* Tween 80 to determine the sink condition of the drug. The devices were placed in a shaking incubator (37 °C, 100 rpm), and the released samples (10 mL) were individually collected and alternated using a fresh buffer for 3 weeks. The amount of PTX released was analyzed via HPLC to determine the release patterns, as previously reported [25,28]. Briefly, an HPLC system fitted with a C18 column (5 μm, 4.6 × 150 mm; SunFire^®^ C18 column, Waters) was used. The absorbance of the released PTX was assessed using a UV detector (Waters 2487) at 228 nm at a flow rate of 1 mL/min. The performance was assessed for 20 min with an injection volume of 10 μL. All the experiments were performed in triplicates.

### 2.4. In Vitro Anticancer Efficacy in Cancer Cells with MDR Effect

MDR-related HCT 15 cells overexpressing p-gp were incubated in a cell culture medium containing RPMI-1640 medium, 10% FBS, and 1% AA at 37 °C in a humidified 5% CO_2_ atmosphere. The anticancer efficacy of PTX itself and that of PTX@TNPs with different drug loads were evaluated using the CCK-8 assay kit as previously described [29,30]. HCT 15 cells were seeded onto 96-well plates at a density of 10,000 viable cells per well and cultured overnight at 37 °C. Next, the medium was replaced with a new one containing PTX (0.1 μg/mL) or PTX@TNPs with different loading amounts of PTX. After treatment for 48 h, the supernatants were replaced with a culture medium containing 10% CCK-8 reagent, and the cells were incubated for an additional 90 min at 37 °C. Finally, the absorbance of the medium in each well was assessed using a microplate spectrophotometer at 450 nm.

### 2.5. Statistical Analysis

All the measurements were performed in triplicates and the data obtained were presented as mean ± standard deviation (SD). Statistical analyses were performed using Student’s *t*-test, and differences were considered significant at *p* < 0.01 (**), and not significant at *p* > 0.05 (#).

## 3. Results and Discussion

### 3.1. Preparation and Characterization of PTX@TNPs

The TNPs, consisting of a PLA core and a Pluronic F127 shell, as biocompatible polymers approved by the FDA, were successfully prepared using the nanoprecipitation method [23]. The TNPs functioned as a good carrier and enhanced the solubilization of the highly lipophilic PTX. Further, via the optimization of the co-solvent ratios for the preparation of PTX@TNPs, the loading amounts and controlled release of PTX were enhanced.

**Figure 2:**
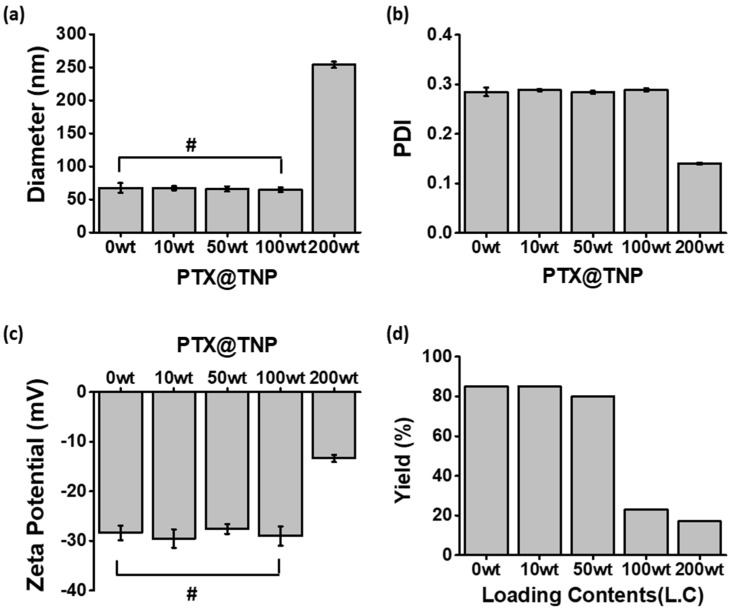
Physicochemical properties of PTX@TNPs with various loading contents, prepared via a single−solvent method using acetone. (**a**) Hydrodynamic diameter, (**b**) polydispersity index (PDI), (**c**) zeta potential, and (**d**) production yield (*n* = 3). ^#^ *p* > 0.05.

As shown in Figure 2a, TNPs were prepared using a single solvent. The hydrodynamic diameter of the TNPs, which was steadily maintained even after the loading of up to 100 wt% of PTX (loading contents compared to PLA amounts in TNP), was 76 ± 1 nm. However, the diameter of PTX@TNPs with 200 wt% of PTX showed a dramatic increase (254 ± 5 nm). Additionally, only the group with 200 wt% of PTX showed a marked difference (PDI, ca. 0.12; zeta potential, ca. −14 mV) compared with that of the bare TNPs (Figure 2b,c). Although PTX@TNPs were well prepared in terms of physicochemical properties up to a PTX loading of 100 wt%, the production yield of PTX@TNPs decreased significantly by ca. 20% after filtration during the preparation process (Figure 2d). This indicates that the solubilization of PTX in TNPs was limited to 50 wt% owing to the low production yield in the group with a PTX loading of 100 wt%.

**Figure 3:**
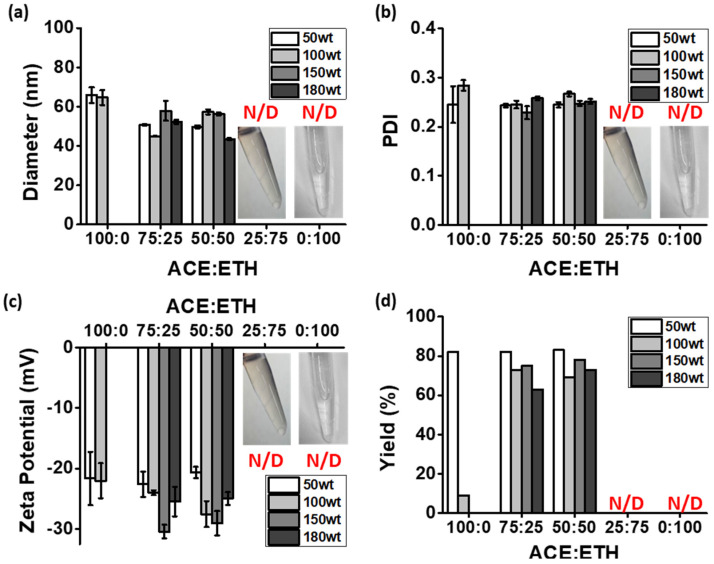
Physicochemical properties of PTX@TNPs prepared by using the co−solvent acetone(ACE)/ethanol(ETH) at various ratios. (**a**) Hydrodynamic diameter, (**b**) polydispersity index (PDI), (**c**) zeta potential, and (**d**) production yield (mean ± SD, *n* = 3; N/D, non–detection).

To improve the solubilization of PTX, we used a co-organic solvent method with acetone and ethanol to prepare the PTX@TNPs. As it was previously reported, the optimization of the co-organic solvent ratio is an important step in achieving enhanced drug loading contents and solubilization in carriers [31]. As shown in Figure 3, the drug loading content in the TNPs increased from 50 to 180 wt% with a high loading efficiency (>95%) when the co-solvent ratios of ACE/ETH increased from 75:25 to 50:50. Further, when co-solvents with over 75% ethanol were used (i.e., at ratios of 25:75 and 0:100), the PLA polymer was not completely dissolved, and TNPs were not formed. Additionally, at co-solvent ratios of 75:25 and 50:50, the physicochemical properties of PTX@TNPs, such as size, PDI, and surface charges, were similar, but the production yield and loading contents of the PTX@TNPs obtained at a 50:50 ratio were higher than those of PTX@TNPs obtained at a co-solvent ratio of 75:25. Furthermore, as shown in Figure S1, the difference in morphology and size between PTX@TNPs (180 wt%) and bare TNPs was not significant. After oral application, the poor water solubility of the drug is the limiting factor of in vivo administration due to its insufficient ability to be diffused into gastrointestinal tract. Thus, attaining high solubilization of poorly water-soluble drugs is a critical element to maximize the incorporation of drugs [32]. The results show that PTX, which has strong lipophilic characteristics, could be highly solubilized in TNPs up to 180 wt% when the optimized co-solvent ratio of 50:50 was employed.

### 3.2. In Vitro Release Behavior of Lipophilic PTX

The release profiles of PTX from PTX@TNPs with different loading amounts (50, 100, 150, and 180 wt%) were examined in PBS containing 0.5% Tween 80 as a sink condition for lipophilic drugs at pH 7.4, as previously reported [33,34]. In order to choose the proper release medium of a drug, phosphate-buffered saline (PBS) is a commonly used medium for the study of the release behavior, but the ratio of nanoparticles to medium might be remarkably low, particularly for hydrophobic drugs. Thus, serum containing PBS can be used as an alternative; however, in order to analyze PTX by HPLC, an additional separation process for sampling should be added, and PTX might be unstable. To overcome this limitation, by adding Tween to PBS, the PBS/Tween can increase the solubility of PTX, and thus no additional process is required for the analysis process, showing good stability.

**Figure 4:**
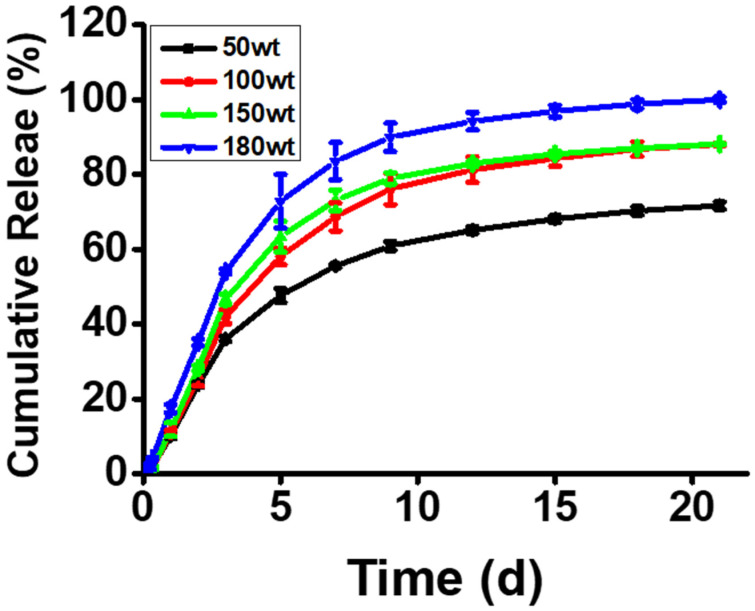
In vitro PTX release profiles from PTX@TNPs with different loading contents in phosphate-buffered saline (PBS, pH 7.4) containing 0.5% (*v*/*v*) Tween 80. The release was performed under gentle stirring and at 37 °C, and the analysis was performed via high-performance liquid chromatography (HPLC).

As shown in Figure 4, the release of PTX from TNPs was well controlled in all groups until three weeks, and the release rate of PTX was accelerated when the loading content of PTX in TNP was increased. According to the loading content increase, the release behavior began with a high initial burst, and the areas occupied by PTX were favored to interact with each other, providing an opportunity for a faster drug release profile [35]. Thus, this result suggests that the release patterns of PTX could be controlled by varying the loading contents in TNPs, leading to a higher release rate and the complete release of the drug from the carrier with a hydrophobic core.

### 3.3. Anticancer Efficacy of PTX@TNPs with Different Drug Loading Contents

The anticancer efficacy of the lipophilic anticancer drug PTX has been limited because it is difficult to control its release rate and ensure the completion of its release profile from the hydrophobic part of the carriers, as it has previously been reported [36,37]. Therefore, PTX@TNPs with different loading contents (50 and 180 wt% of PTX) were compared to assess the enhanced anticancer efficacy against p-gp-overexpressing HCT 15 cells under different solubilization and controlled PTX release conditions.

**Figure 5:**
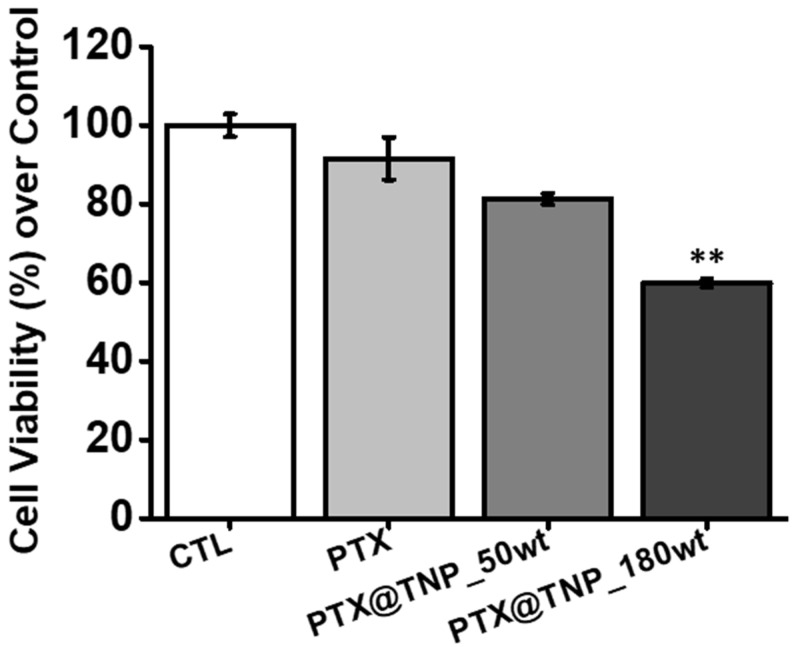
Anticancer efficacy of PTX@TNPs with different PTX loading amounts in the inhibition of cell proliferation in HCT 15 cells. Cells were incubated with free PTX or PTX@TNPs (50 wt% and 180 wt%) containing 0.1 μg/mL PTX for 48 h. The cell viability was analyzed using a cell counting kit-8 (CCK-8) assay, and it is presented as the mean ± SD of at least triplicate experiments. ** *p* < 0.01, comparison between the free drug and PTX@TNPs.

As shown in Figure 5, only the anticancer effect of PTX was not significant compared to CTL (no drug treatment) due to the MDR effect in the colon cancer cells, as expected. However, notably, PTX@TNPs showed a significant improvement in the anticancer effect compared with the free drug. In particular, the TNP group with a higher loading content of PTX (180 wt%) showed a more than 1.5-fold superior anticancer effect compared with the free drug.

Additionally, TNPs with a Pluronic F127 shell possibly show a synergistic anticancer effect given that the Pluronic component has the potential to act as a p-gp inhibitor [38,39,40]. In a previous report, the in vitro anticancer efficacy of PTX-loaded Pluronic P123/F127 mixed micelles (PF-PTX) was higher compared to free PTX, ranging from 0.005 to 1 μg/mL. Therefore, the Pluronic might act as a chemosensitizer and enhance the antitumor effect of PTX through blockage of P-gp by ATP deficiency in MDR tumor cells [41]. In addition, this implies that the high solubilization and high release rate of lipophilic PTX resulting from the use of Pluronic-based TNPs contributed significantly to the enhanced anticancer efficacy in MDR-related cancer cells. Therefore, TNPs could be used as promising delivery carriers for the improved solubilization and controlled release of various lipophilic anticancer drugs to the end of realizing effective anticancer therapy in malignant tumors, especially in MDR-related tumors.

## 4. Conclusions

In this study, we demonstrated the improved solubilization and controlled release of a lipophilic anticancer drug based on the use of TNPs as carriers. PTX@TNPs with different PTX loading contents were successfully prepared via a simple nanoprecipitation method, without chemical conjugation, using varying ratios of co-organic solvents. The solubilization, loading content, and production yields of PTX in the TNPs were then optimized by analyzing the physicochemical properties of the prepared complex, PTX@TNP. Thus, it was observed that PTX@TNPs showed a faster release behavior as the PTX loading content increased. Further, PTX@TNPs with a higher loading content of PTX (180 wt%) resulted in an over 1.5-fold higher synergistic anticancer effect in p-gp-overexpressing colorectal cancer cells compared with the free drug. This observation could be attributed to the higher solubilization and controlled release of PTX in TNPs. Further, these results suggest that TNPs have a strong potential as carriers for the improvement in the solubilization of lipophilic anticancer drugs; this has significance in the realization of successful anticancer therapies.

## Data Availability

Not applicable.

## Supplementary Materials

The following are available online at https://www.mdpi.com/article/10.3390/pharmaceutics13081150/s1, Figure S1: TEM images of (**a**) TNP and (**b**) PTX@TNP (180 wt). The scale bar indicates 200 nm.

## References

[1] Bray F., Ferlay J., Soerjomataram I., Siegel R.L., Torre L.A., Jemal A. (2018). Global cancer statistics 2018: GLOBOCAN estimates of incidence and mortality worldwide for 36 cancers in 185 countries. CA Cancer J. Clin..

[2] Miller K.D., Leticia N., Angela B.M., Julia Y.R.K., Catherine M.A., Ahmedin J., Joan L.K., Rebecca L.S. (2019). Cancer treatment and survivorship statistics, 2019. CA Cancer J. Clin..

[3] Chabner B.A., Roberts T.G. (2005). Timeline: Chemotherapy and the war on cancer. Nat. Rev. Cancer.

[4] Kumar P., Raza K., Kaushik L., Malik R., Arora S., Katare O.P. (2016). Role of colloidal drug delivery carriers in taxane-mediated chemotherapy: A review. Curr. Pharm. Des..

[5] Wang F., Zhang D., Zhang Q., Chen Y., Zheng D., Hao L., Duan C., Jia L., Liu G., Liu Y. (2011). Synergistic effect of folate-mediated targeting and verapamil-mediated P-gp inhibition with paclitaxel-polymer micelles to overcome multi-drug resistance. Biomaterials.

[6] Naderinezhad S., Amoabediny G., Haghiralsadat F. (2017). Co-delivery of hydrophilic and hydrophobic anticancer drugs using biocompatible pH-sensitive lipid-based nano-carriers for multidrug-resistant cancers. RSC Adv..

[7] Palmeira A., Sousa E., Vasconcelos M.H., Pinto M.M. (2012). Three decades of P-gp inhibitors: Skimming through several generations and scaffolds. Curr. Med. Chem..

[8] Nigam S.K. (2014). What do drug transporters really do?. Nat. Rev. Drug. Discov..

[9] Esim O., Sarper M., Ozkan C.K., Oren S., Baykal B., Savaser A., Ozkan Y. (2020). Effect simultaneous delivery with P-glycoprotein inhibitor and nanoparticle administration of doxorubicin on cellular uptake and in vitro anticancer activity. Saudi Pharm. J..

[10] Koziara T.M., Whisman T.R., Tseng M.T., Mumper R.J. (2006). In-vivo efficacy of novel paclitaxel nanoparticles in paclitaxel-resistant human colorectal tumors. J. Control. Release.

[11] Jia L., Li Z., Shen J., Zheng D., Tian X., Guo H., Chang P. (2015). Multifunctional mesoporous silica nanoparticles mediated co-delivery of paclitaxel and tetrandrine for overcoming multidrug resistance. Int. J. Pharm..

[12] Fellner F., Bauer B., Miller D.S., Schaffrik M., Fankhänel M., Spruss T., Bernhardt G., Graeff C., Färber L., Gschaidmeier H. (2002). Transport of paclitaxel (Taxol) across the blood–brain barrier in vitro and in vivo. J. Clin. Investig..

[13] Mickisch G.H., Pai L.H., Gottesman M.M., Pastan I. (1992). Monoclonal antibody MRK16 reverses the multidrug resistance of multidrug-resistant transgenic mice. Cancer Res..

[14] Singla A.K., Garg A., Aggarwal D. (2002). Paclitaxel and its formulations. Int. J. Pharm..

[15] Lee S.C., Kim C., Kwon I.C., Chung H., Jeong S.Y. (2003). Polymeric micelles of poly(2-ethyl-2-oxazoline)-block-poly(epsilon-caprolactone) copolymer as a carrier for paclitaxel. J. Control. Release.

[16] Jin K.T., Lu Z.B., Chen J.Y., Liu Y.Y., Lan H.R., Dong H.Y., Yang F., Zhao Y.Y., Chen X.Y. (2020). Recent Trends in Nanocarrier-Based Targeted Chemotherapy: Selective Delivery of Anticancer Drugs for Effective Lung, Colon, Cervical, and Breast Cancer Treatment. J. Nanomater..

[17] Jiang M., Zhang R., Wang Y., Jing W., Liu Y., Ma Y., Sun B., Wang M., Chen P., Liu H. (2017). Reduction-sensitive paclitaxel prodrug self-assembled nanoparticles with tetrandrine effectively promote synergistic therapy against drug-sensitive and multidrug-resistant breast cancer. Mol. Pharm..

[18] Afrooz H., Ahmadi F., Fallahzadeh F., Mousavi-Fard S.H., Alipour S. (2017). Design and characterization of paclitaxel-verapamil co-encapsulated PLGA nanoparticles: Potential system for overcoming P-glycoprotein mediated MDR. J. Drug Deliv. Sci. Technol..

[19] Patel N.R., Rathi A., Mongayt D., Torchilin V.P. (2011). Reversal of multidrug resistance by co-delivery of tariquidar (XR9576) and paclitaxel using long-circulating liposomes. Int. J. Pharm..

[20] Shukla A.J., Price J.C. (1991). Effect of drug loading and molecular weight of cellulose acetate propionate on the release characteristics of theophylline microspheres. Pharm. Res..

[21] Chang R.K., Price J., Whitworth C.W. (1987). Control of drug release rate by use of mixtures of polycaprolactone and cellulose acetate butyrate polymers. Drug Dev. Ind. Pharm..

[22] Chen H., Lui Y.S., Zhao J., Xu L., Tan L.P. (2018). Effect of solvent composition of electrospun PLGA fibers on paclitaxel release. Mater. Technol..

[23] Choi W.I., Kamaly N., Riol-Blanco L., Lee I.H., Wu J., Swami A., Vilos C., Yameen B., Yu M., Shi J. (2014). A solvent-free thermosponge nanoparticle platform for efficient delivery of labile proteins. Nano Lett..

[24] Lee J.S., Hwang Y., Oh H., Kim S., Kim J.-H., Lee J.-H., Shin Y.C., Tae G., Choi W.I. (2019). A novel chitosan nanocapsule for enhanced skin penetration of cyclosporin A and effective hair growth in vivo. Nano Res..

[25] Choi J.S., Park J.S. (2016). Effects of paclitaxel nanocrystals surface charge on cell internalization. Eur. J. Pharm. Sci..

[26] Hu H., Wang B., Lai C., Xu X., Zhen Z., Zhou H., Xu D. (2019). iRGD-paclitaxel conjugate nanoparticles for targeted paclitaxel delivery. Drug Dev. Res..

[27] Mauro P.P.D., Borrós S. (2014). Development of high drug loaded and customizing novel nanoparticles for modulated and controlled release of paclitaxel. Pharm. Res..

[28] Sohn J.S., Yoon D.S., Sohn J.Y., Park J.S., Choi J.S. (2017). Development and evaluation of targeting ligands surface modified paclitaxel nanocrystals. Mater. Sci. Eng. C Mater. Biol. Appl..

[29] Yu N., Li J., Zhang Y., Ding D., Li X., Xu H. (2020). Superior antitumor effect of self-assembly supramolecular paclitaxel nanoparticles. RSC Adv..

[30] Huang Y., Sun R., Luo Q., Wang Y., Zhang K., Deng X., Zhu W., Li X., Shen Z. (2016). In situ fabrication of paclitaxel-loaded core-crosslinked micelles via thiol-ene “click” chemistry for reduction-responsive drug release. J. Polym. Sci. Part A Polym. Chem..

[31] Obeidat W.M., Price P.C. (2006). Preparation and evaluation of Eudragit S 100 microspheres as pH-sensitive release preparations for piroxicam and theophylline using the emulsion-solvent evaporation method. J. Microencapsul..

[32] Hu J., Johnston K.P., Williams R.O. (2004). Nanoparticle Engineering Processes for Enhancing the Dissolution Rates of Poorly Water Soluble Drugs. Drug. Dev. Ind. Pharm..

[33] Abouelmagd S.A., Sun B., Chang A.C., Ku Y.J., Yeo Y. (2015). Release Kinetics Study of Poorly Water-Soluble Drugs from Nanoparticles: Are We Doing It Right?. Mol. Pharm..

[34] Jain S., Kumar D., Swarnakar N.K., Thanki K. (2012). Polyelectrolyte stabilized multilayered liposomes for oral delivery of paclitaxel. Biomaterials.

[35] Hsu S.-T., Yao Y.L. (2013). Effect of drug loading and laser surface melting on drug release profile from biodegradable polymer. J. Appl. Polym. Sci..

[36] Mu J., Zhong H., Zou H., Liu T., Yu N., Zhang X., Xu Z., Chen Z., Guo S. (2020). Acid-sensitive PEGylated paclitaxel prodrug nanoparticles for cancer therapy: Effect of PEG length on antitumor efficacy. J. Control. Release.

[37] Levit S.L., Yang H., Tang C. (2020). Rapid Self-Assembly of Polymer Nanoparticles for Synergistic Codelivery of Paclitaxel and Lapatinib via Flash NanoPrecipitation. Nanomaterials.

[38] Minko T., Batrakova E.V., Li S., Li Y., Pakunlu R.I., Alakhov V.Y., Kabanov A.V. (2005). Pluronic block copolymers alter apoptotic signal transduction of doxorubicin in drug-resistant cancer cells. J. Control Release.

[39] Kabanov A.V., Batrakova E.V., Alakhov V.Y. (2002). Pluronic block copolymers for overcoming drug resistance in cancer. Adv. Drug Deliv. Rev..

[40] Batrakova E.V., Li S., Vinogradov S.V., Alakhov V.Y., Miller D.W., Kabanov A.V. (2001). Mechanism of pluronic effect on P-glycoprotein efflux system in blood–brain barrier: Contributions of energy depletion and membrane fluidization. J. Pharmacol. Exp. Ther..

[41] Wei Z., Yuan S., Chen Y., Yu S., Hao J., Luo J., Sha X., Fang X. (2010). Enhanced antitumor efficacy by Paclitaxel-loaded Pluronic P123/F127 mixed micelles against non-small cell lung cancer based on passive tumor targeting and modulation of drug resistance. Eur. J. Pharm. Biopharm..

