# Supplementary material for: High Solubilization and Controlled Release of Paclitaxel Using Thermosponge Nanoparticles for Effective Cancer Therapy
Source: Pharmaceutics. 2021 Jul 27;13(8):1150. doi: 10.3390/pharmaceutics13081150 (PMC8398058; doi:10.3390/pharmaceutics13081150)
Supplement: Supplementary file 1 [file pharmaceutics-13-01150-s001.zip › pharmaceutics-1304381-supplementary.pdf]

pharmaceutics

Supplementary Materials: High Solubilization and Controlled Release of Paclitaxel Using Thermosponge Nanoparticles for Effective Cancer Therapy

Jin Sil Lee, Hyeryeon Oh, Daekyung Sung, Jin Hyung Lee, Won Il Choi

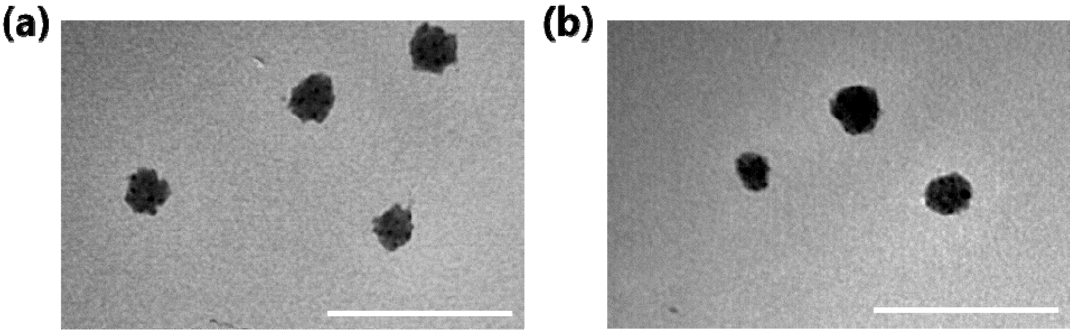

Figure S1. TEM images of (**a**) TNP and (**b**) PTX@TNP (180 wt). The scale bar indicates 200 nm.
